# Supplementary material for: Nanobody-Based EGFR-Targeting Immunotoxins for Colorectal Cancer Treatment
Source: Biomolecules. 2023 Jun 26;13(7):1042. doi: 10.3390/biom13071042 (PMC10377705; doi:10.3390/biom13071042)
Supplement: Supplementary file 1 [file biomolecules-13-01042-s001.zip › biomolecules-2417327-supplementary.pdf]

Supplementary Materials: New nanobody-based EGFR-targeting immunotoxins for colorectal cancer treatment.

Narbona J. et al

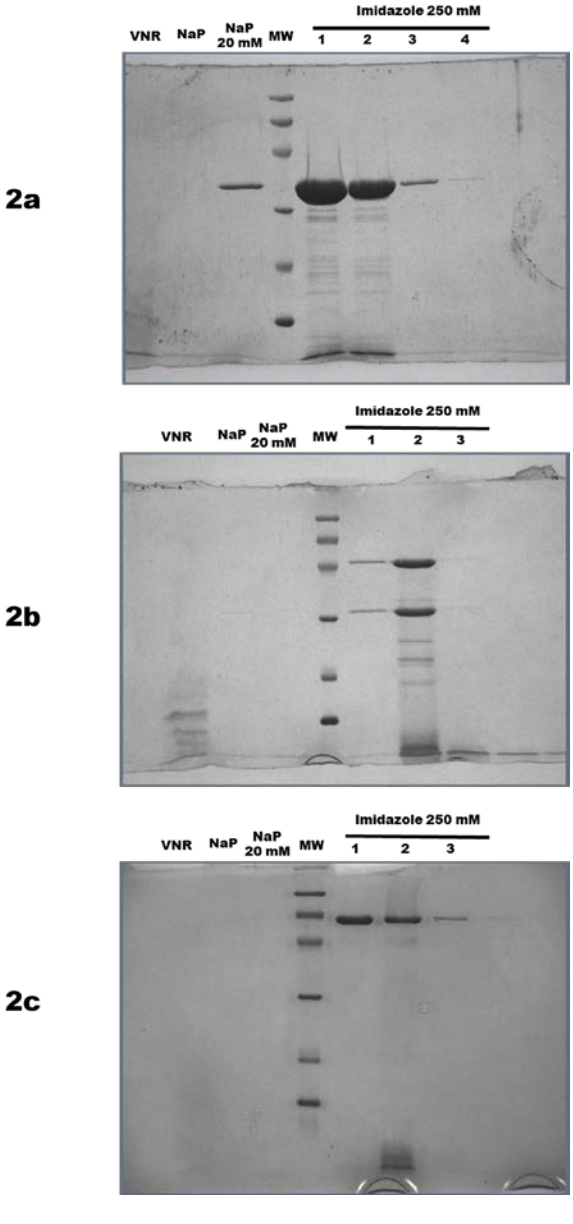

Figure S1. Original SDS-PAGE of purified nanoimmunotoxins. Original full-length gels from Figure 2 are presented. Coomassie blue stained SDS-PAGE analysis of the different fractions obtained during the Ni^{2+}-NTA affinity chromatography of V_{HH}EGFRαS (**a**), TriV_{HH}EGFRαS (**b**) and BsITXαSDI (**c**). Lines shown correspond to: MW, prestained molecular weight standard (KDa); VNR, nor retained fraction; NaP, washed fraction eluted with sodium phosphate buffer; NaP 20 mM, washed fraction eluted with sodium phosphate buffer containing imidazole 20 mM; and different 1 ml fractions eluted with 250 mM imidazole. Images were acquired and analyzed using the Quantity One 1-D analysis software (BioRad).

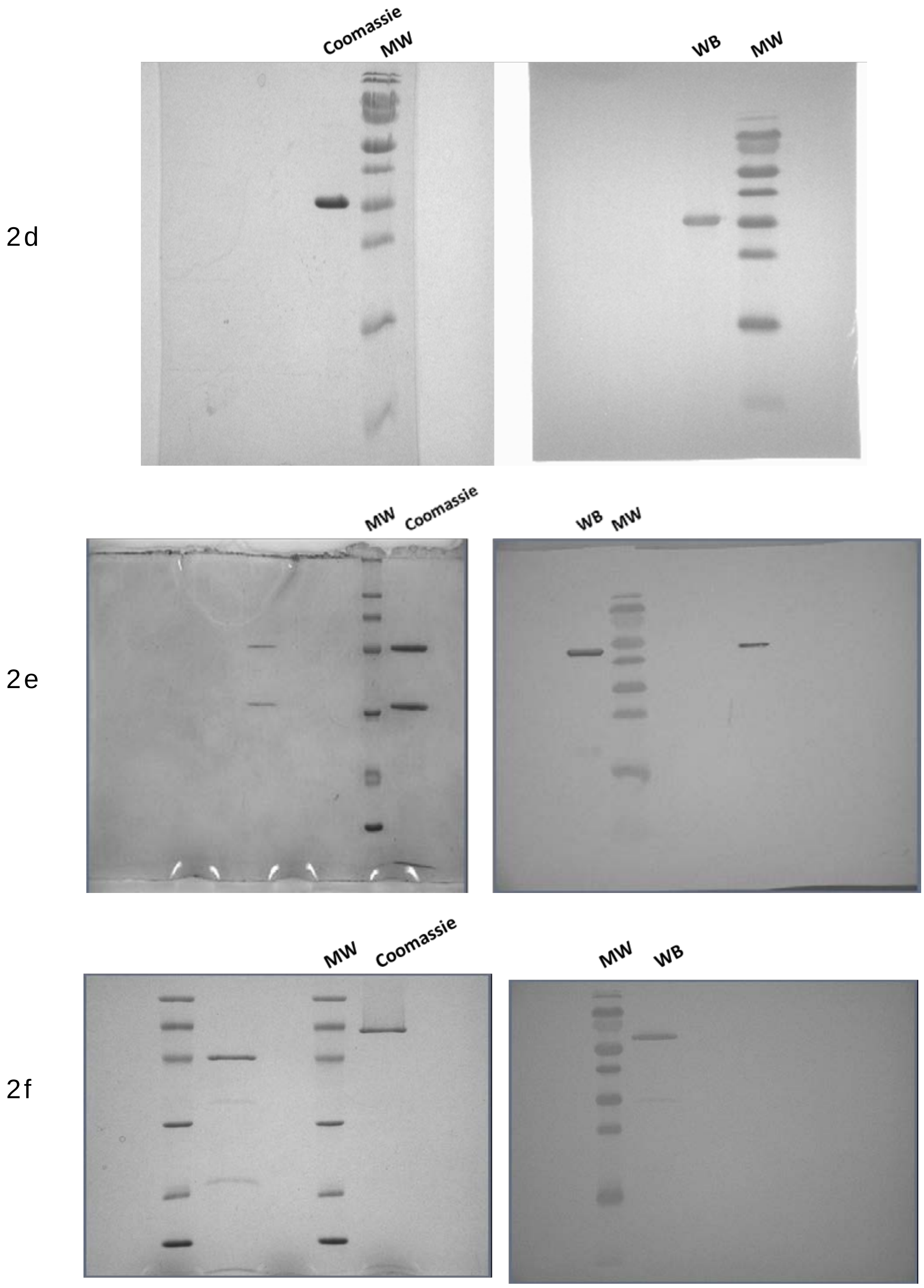

Figure S2. SDS-PAGE and Western Blot analysis of all purified nanoimmunotoxins. Original full-length gels and western blots from Figure 2d, 2e and 2f are presented. In all cases lines corresponding to information appeared in Figure 2 are indicated. SDS-PAGE followed by Coomassie blue staining (left) and Western Blot analysis (right) of the purified final fraction of V_{HH}EGFRαS (**d**), TriV_{HH}EGFRαS (**e**) and BsITXαSDI (**f**). Western blot analysis was carried out using rabbit anti-α-sarcin serum. MW corresponds to prestained molecular weight standards. Images were acquired and analyzed by the Quantity One 1-D analysis software (BioRad).

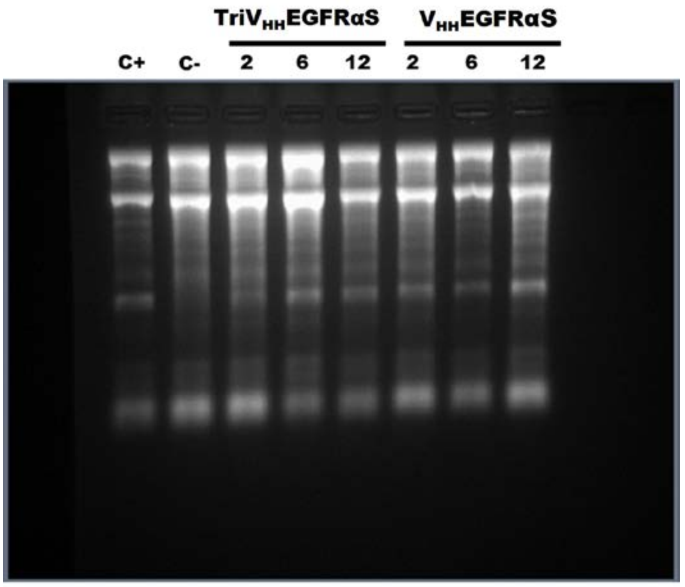

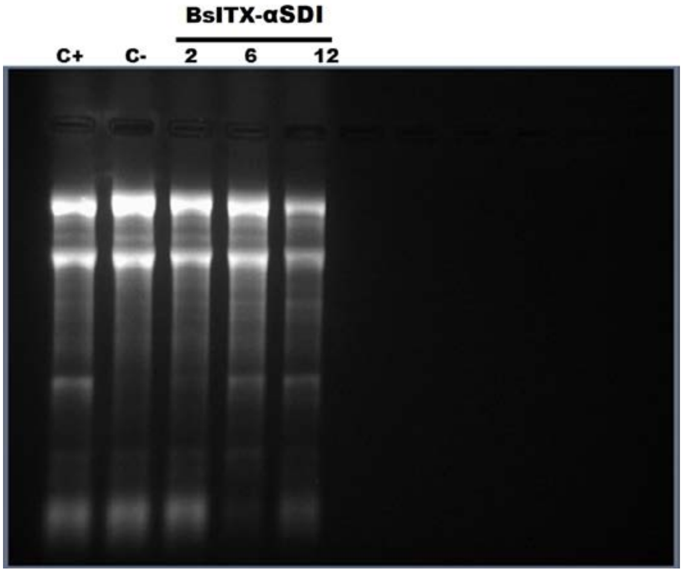

Figure S3. ***In vitro* functional characterization.** Original full-length gels from Figure 3 are presented. Ribonucleolytic activity of the toxic domain of V_{HH}EGFRαS and TriV_{HH}EGFRαS (**a**) and BsITXαSDI (**b**). The arrow indicates the release of the α-fragment, produced by the cleavage of the SRL due to the α-sarcin. In both gels, 2, 6 and 12 pmoles of all three nanoimmunotoxins were assayed. C+ represents 2 pmoles of fungal wild-type α-sarcin, whereas in C-, the protein sample was replaced by buffer. Images were acquired and analyzed using the Quantity One 1-D analysis software (BioRad).
